# Immune age is correlated with decreased TCR clonal diversity and antibody response to SARS-CoV-2

**DOI:** 10.1038/s41598-025-04736-4

**Published:** 2025-06-06

**Authors:** Merlin Davies, Hubert Denise, Michael Day, Sian M Henson, Chris J Scotton, Lorna W. Harries

**Affiliations:** 1 Department of Clinical and Biomedical Sciences, RILD building, Royal Devon and Exeter University Hospital, University of Exeter, Barrack Road, Exeter, EX2 5DW UK; 2 SENISCA Ltd, RILD Building, Royal Devon and Exeter University Hospital, Barrack Road, Exeter, EX2 5DW UK; 3 Molecular Diagnostics, Royal Devon and Exeter University Hospital, Barrack Road, Exeter, EX2 5DW UK; 4 Translational Medicine and Therapeutics, The London School of Medicine and Dentistry, William Harvey Research Institute, Queen Mary University of London, Barts, London, EC1M 6BQ UK

**Keywords:** Immune age, Senescence, SARS-CoV-2, Immune function, T cell, Cell biology, Immunology, Molecular biology, Medical research, Molecular medicine

## Abstract

Immune response to infection or vaccination is compromised with age. We aimed to examine associations between immune senescence, T and B cell clonal diversity and immunoglobulin G secretion in response to immune challenge in isolated peripheral blood mononuclear cells (PBMC) from people of different chronological ages. We isolated PBMC from 49 individuals categorised into < 35 years and > 60 years age groups. Cells were then challenged with recombinant SARS-CoV-2 spike protein or vehicle and IMMAX score was calculated for each sample from flow cytometry. Antibody response was assessed using the proxy of IgG secretion and T cell receptor and immunoglobulin framework region recombination was determined by clonality studies. We observed that individuals aged > 60 years demonstrated a higher immune ‘age’ as calculated by IMMAX score (0.75 compared with 0.48 for individuals aged < 35 years; p = < 0.0001). Immune age negatively correlated with IgG responsivity in older individuals with recent prior exposure to SARS-CoV-2 (b = -0.01; *p* = 0.05). Higher immune age was also negatively correlated with TCR Vd + Jd receptor diversity regardless of immune challenge (b = -0.02; p = < 0.0001 and b = -0.02; r^2^ = 0.0.35; p = < 0.0001 for control and exposed samples respectively). Our data demonstrate that PBMC samples from older people display a higher cellular immune age and attenuation of immune response. This suggests that future treatments targeting cellular ageing of immune cells may be a useful avenue for investigation to improve immune function in older people.

**Supplementary Information:**

The online version contains supplementary material available at 10.1038/s41598-025-04736-4.

## Introduction

Older people are at increased risk of adverse outcomes following exposure to infectious agents and demonstrate impaired responses to vaccination. In the SARS-CoV-2 pandemic, older individuals exposed to SARS-CoV-2 vaccines demonstrated diminished immune responses, lower antibody titres, increased mortality and reduced duration of protection relative to younger people^1^. Furthermore, reduced neutralising antibody production against SARS-CoV-2 vaccines has been linked to age-related frailty in older individuals^2^. This observation is not limited to SARS-CoV-2; vaccinations against other highly mutable viruses such as influenza also present decreased efficacy with age^3^.

Senescence of the immune system (immunosenescence) is an important contributor to the decline in immune function with advancing age^4^. Senescent cells accumulate in all tissues and can be induced by a variety of cellular stimuli such as replicative or metabolic stress, DNA damage or the paracrine activity of neighbouring senescent cells^5^. Cellular senescence is defined by cell cycle arrest, senescence-associated beta galactosidase (SA-b-Gal) positivity, elevated DNA damage and characteristic morphological and functional changes^6^. Senescent cells accumulate in the body during the ageing process and is now widely accepted as a driver of systemic ageing^7^ and its associated diseases^8–10^. Senescent cells secrete the senescence-associated secretory phenotype (SASP), a mixture of proinflammatory cytokines, chemokines and growth factors which contributes to the chronic inflammation of ageing^11^. The SASP has been suggested to have evolved to signal clearance of aged or dysfunctional cells from tissues and organs via the immune system^11^ and is tissue specific. Several factors including IL-6, IL-8, IL1a/b and IFN-γ have however been described to be present in multiple tissues^12^.

Characteristic changes in immune cell compartments occur with age. Production of naïve T cells is limited in older individuals, leading to an altered ratio of CD4^+^ and CD8^+^ T cells^13^. The diversity of T cell and B cell responses to immune challenge also becomes compromised in ageing individuals. Restriction of the clonal diversity generated by rearrangement of the T cell receptor (TCR) alpha, beta and delta genes has been described^14^, as has reduced diversity of immunoglobulin heavy and light chain gene rearrangements^15^. These features are necessary to generate a polyclonal wide-ranging immune response^16^. A reduced immune repertoire leads to compromised functionality in response to infectious agents, as well as altered cell-cell communication within the adaptive immune system^17,18^. Clonal diversity of the immunoglobulin and T cell receptor genes is very important for the removal of senescent cells, which requires a wide repertoire of clones to recognise a wide variety of senescence-associated antigens^19^. The reduced clonal diversity of immune cells, and the resulting restriction of T cell and antibody responses that occurs with ageing could therefore contribute to the accumulation of senescent cells. Senescent dermal fibroblasts express the non-classical HLA marker HLA-E, which together with highly differentiated CD8 + cells inhibits immune responses to senescent cells^20^.

In this study, we aimed to investigate the interplay between immune age as measured by IMMAX and changes in immunoglobulin (IgG) responsivity after immune challenge. We also assessed the relationship between immune age and T or B cell clonal diversity in community-living older people in an experimental system completely devoid of any animal derived biomaterials. We first assessed the changes in immune cell populations that occurs during ageing in isolated human peripheral blood derived mononuclear cells (PBMC) from older (> 60 years) and younger (< 35 years) study participants. We then determined the ‘biological’ immune age of the samples using IMMAX and assessed the relationship between immunological age and IgG response to challenge with recombinant SARS-CoV-2 spike proteins in individuals of different ages. Finally, we characterised T cell receptor (TCR) and IgH immunoglobulin framework region (FR) gene rearrangement diversity in immune cells from older and younger individuals and assessed the relationship between these parameters and biological immunological age derived by IMMAX. IMMAX is an adapted model for defining immunological age using large datasets, aimed at overcoming population immunological heterogeneity across biological age^21^.

A better understand of the functional basis behind interactions between a senescent immune system and response to infection or immunisation may allow the design of drugs targeted to restoration or improvement of immune function and improvement in vaccine response in at risk groups.

## Methods

### Sample collection and cohort demographics

The sample set consisted of 49 consented individuals classified as either ‘younger’ (≤ 35 years) or ‘older’ (≥ 60 years). Samples were collected with informed consent on the Royal Devon and Exeter Universities Foundation Trust site between 01/04/2022 and 30/11/2022 under ethical permission granted by the University of Exeter Medical School Health and Community Sciences ethics committee (study number 511048). All methods were carried out in accordance with relevant guidelines and regulations. We excluded individuals with a history of autoimmune disorders or active cancers but had no other exclusion criteria. All individuals sampled had not experienced a SARS-CoV-2 infection or vaccination within 4 weeks prior to blood sampling. Participant demographics are presented in Table 1. Participants were also asked to complete a short questionnaire regarding basic demographics and SARS-CoV-2 infection/vaccine history. Ethical permission was obtained from the University of Exeter’s College of Medicine and Health Ethics Committee (ethics number: 511048). Samples were collected into Lithium heparin tubes (Fisher Scientific, Loughborough, UK), and peripheral blood mononuclear cells (PBMC) were isolated using Sepmate-50 Isolation tubes according to manufacturer’s instructions (Stemcell Technologies, 85450, Vancouver, Canada) prior to resuspension in 900 µL RPMI cell medium (Fisher Scientific, Loughborough, UK) and 10% DMSO for cryopreservation ahead of experimentation.

**Table 1:** Demographic and anthropometric characterisation of participants. A total of 49 individuals participated in our study, stratified into two age groups (< 35 years and > 65 years).

Patient Characteristics (*N* = 49)	
N (< 35 years)	22
N (> 60 years)	27
% Male (< 35 years)	50%
% Male (> 60 years)	29.6%
Mean and SD age (< 35 years)	27 (3.95)
Mean and SD age (> 60 years)	71 (6.54)
Mean and SD T cell count/mL (< 35 years)	5.7 × 10^5^ (4.1 × 10^5^)
Mean and SD T cell count/mL (> 60 years)	5.7 × 10^5^ (3.0 × 10^5^)
% SARS-CoV-2 vaccinated (< 35 years)	95.5%
% SARS-CoV-2 vaccinated (> 60 years)	96.3%
% COVID Infection last 6 months (< 35 years)	36.4%
% COVID Infection last 6 months (> 60 years)	16.5%

### Cell culture methods

Cells were resuspended at a density of 1 × 10^6^ cells/mL into 24 well plates in RPMI cell culture media (Fisher Scientific, Loughborough, UK)) supplemented with 10% GroPro/Human Serum (GroPro, SER-HPL-GROPRO, Zen-Bio, Cambridge, UK/Merck, H4522, Darmstadt, Germany), 1% Penicillin-Streptomycin (P/S) (Fisher Scientific, 15070063, Hampton, USA) and T-cell activation components (Ionomycin (10 µg/ml) (Alfa Aesar, J62448.M, Massachusetts, USA), PMA (100 ng/ml) (Alfa Aesar, J63916.M, Massachusetts, USA), IL-2 (100 ng/µL) (Miltenyi Biotec, 130-097-743, North Rhine-Westphalia, Germany) and α-CD3 (0.1 µg/ml) (Miltenyi Biotec, 130-122-282, North Rhine-Westphalia, Germany) as previously described^22^. For mimicry of infection, cells were incubated for 48 h to acclimatise. SARS-CoV-2 antigen stimulation was performed by the addition of 100 ng/mL of recombinant SARS-CoV-2 spike protein of subtype Omicron B.1.1.529 (Biolegend, 792906, California, USA) in three replicates for a further 48 h. At the end of culture, cells were harvested for immediate extraction of RNA/DNA and media were harvested by centrifugation and stored at −80 °C until use.

### Determination of immune age

PBMC samples were fixed in 100 µL of 2% Paraformaldehyde (PFA) and permeabilised using 0.05% Triton prior to flow cytometry assessment of T cell and senescence markers. T cell senescence was inferred using a panel of previously described intracellular and extracellular T cell and T cell senescence on the Cytek Aurora Spectral Flow Cytometry Platform (Cytek Bio, California, USA). Identity and concentration of antibodies used, suppliers, fluorochromes and fluorescent spectra properties are described in supplementary Table 1. KLRG1, CD28, CCR7, NKG2 A, NKG2D, CD57, FOXP3, CD24 and CD25 expression was evaluated across the 4 major T cell subsets in both CD4^+^ and CD8^+^ T cells and the distribution of markers assessed was used to infer the 4 major T cell subsets (naïve, effector memory (EM), central memory (CM) and terminally differentiated effector memory cells re-expressing CD45RA (EMRA) in both CD4^+^ and CD8^+^ T cells. Gating into naïve, EM, CM and EMRA T cell subsets was performed using CD27 and CD45RA markers and data were analysed in FlowJo (BD, New Jersey, USA). Statistical significance of observed changes was assessed using multiple t tests in Graphpad Prism (Dotmatics, Boston, USA). Immune age was calculated using IMMAX scoring, which was generated using an adapted script from Brode et al.^21^. Raw data for 7 immune cell lines (Naive_CD4^+^, EM_CD4^+^, EM_CD8^+^, EMRA_CD8^+^, CD28^−^_CD8^+^, CD57^+^_CD8^+^ and FOXP3^+^_CD4^+^) was normalised to the mean total T cell frequency; 70% of initial total PBMC cell counts, as previously reported^23^. The raw cell frequency data for 7 immune cell lines (Naive_CD4^+^, EM_CD4^+^, EM_CD8^+^, EMRA_CD8^+^, CD28^−^_CD8^+^, CD57^+^_CD8^+^ and FOXP3^+^_CD4^+^) for 49 donors was normalised to the mean frequency to total T cells (70% of initial total PBMC cell counts, as previously reported)^23^. The IMMAX algorithm^21^ performed Principal Component Regression to derive models using the normalised frequencies and selected the lowest-error model to calculate the IMMAX score as immune age metric. Adapted script is available here: https://github.com/DrMerlinDavies/IMMAX2024/blob/main/R_Script.

### ‘Sandwich’ Enzyme-linked Immunosorbent Assays for antibody quantification

Enzyme-linked Immunosorbent assays (ELISAs) were used to assay changes in IgG production as a proxy for antibody response to recombinant SARS-CoV-2 spike exposure. Sandwich assays were prepared using Immulon 2 Hb flat-bottom 96-well plates coated with anti-IgG antibody (SinoBiological, 10702-MM01 T-H, China) diluted in 1x coating buffer (Bio-Rad, Watford, UK). Plates were then blocked with synthetic blocking buffer (ECO-TEK Synthetic Blocking Buffer, 4250-T, 2BScientific, Kidlington, UK) and washed three times with 300 µL wash buffer (PBS + 0.05% Tween) prior to use. A two-fold serial dilution standard curve of recombinant IgG antibody (SinoBiological, 68090-MM04-H, China) was established, starting at 225 ng/mL with 7 subsequent dilutions in wash buffer (PBS + 0.05% Tween) for sample quantification. The secondary antibody used was an HRP conjugated anti-IgG antibody (SinoBiological, 10702-MM01 T-H, China), used at a 1 in 10,000 dilution. 100 µL of TMB substrate solution (Merck, ES001-100 mL, Darmstadt, Germany) was added to each well and the plate incubated at room temperature in the dark. Following this, 100 µL of STOP solution (Merck, S5689, Darmstadt, Germany) was added to each well. Signal was quantified on the Pherastar™ platform at 650 nm to generate well and sample specific absorbance readings, which were quantified relative to the standard curve. Changes in IgG levels refers to the difference in IgG measurement in the untreated cells (baseline immune activation without spike protein) and after treatment of cells from the same sample with recombinant SARS-CoV-2 spike protein were then assessed using repeated measures ANOVA by GraphPad Prism (Dotmatics, Boston, USA). To determine whether the immune age of PBMC samples has any impact on antibody production, we also assessed the relationship between IMMAX score and change in IgG secretion by linear regression. Data were assessed in the population but also stratified according to prior participant self-reported, positive testing exposure to recombinant SARS-CoV-2 spiked protein.

## Assessment of Immunoglobulin and T cell receptor gene rearrangements

Older individuals often display restricted B cell and T cell immunoglobulin and T cell receptor gene rearrangements. DNA was extracted using 0.3 v/v of TRI Reagent (Invitrogen, 11312940, US), according to manufacturer’s instructions and used for analysis of immunoglobulin heavy chain and T cell receptor beta and delta gene rearrangement patterns. Prior to use in analysis, the pH of the sample was adjusted to 8.0 with 0.1 M HEPES buffer. DNA quality and quantity were assessed using a Thermo Scientific™ Nanodrop 8000 Spectrophotometer (Waltham, USA). B cell and T cell clonality patterns for each sample were assessed in triplicate by analysis of the immunoglobulin heavy chain FR2B and FR3B genes and the T cell receptor TCR-β and TCR-δ genes respectively. B cell clonality primers were as described in^24^, and were procured from Sigma-Aldrich (Missouri, USA). B cell clonality reactions were carried out in three biological replicates in 96 well plates. B cell clonality reaction volume was 10 µL and contained 1 µL water, 1.5 µL PCR buffer (10x), 0.9 µL MgCl_2_, 1.5 µL dNTPs and 1 µL of each primer (500ng/µL) and 0.1 µL Amplitaq Gold polymerase (5U/µL). Cycling conditions were 94 ⁰C for 7 min; then 35 cycles of 94 ⁰C for 45 s, 60 ⁰C for 45 s, and 72 ⁰C for one minute and 30 s; with the final step being 72 ⁰C for 10 min. T cell clonality was measured using the Invivoscribe Clonality TCRβ and δ Assays (Invivoscribe, 92050011 and 92060011 respectively, California, USA). Each assay contains different primer combinations for VDJ recombination clones within the TCRβ and δ subunits. Each reaction well contained 100ng DNA sample, 10 µL reaction mix and 0.1 µL Amplitaq Gold polymerase (5U/µL). The thermal cycler profile was 94 ⁰C for 10 min; then 35 cycles of 94 ⁰C for 1 min, 60 ⁰C for 1 min, 72 ⁰C for one minute; with the final step being 72 ⁰C for 10 min. PCR products were diluted and mixed with 8.9 µL Hi-Di Formamide and 0.1 µL GS500 ROX. Samples were then analysed on the Applied Biosystems 3730 Genetic Analyser using Microsatellite default settings. Associations were assessed using a multiple comparisons ANOVA. To determine whether the immune age of PBMC samples has any impact on immunoglobulin heavy chain framework region or T cell receptor (TCR) beta or gamma gene rearrangement, we also assessed the relationship between IMMAX score and change in IgG secretion by linear regression.

## Results

### Older people have an altered CD8/CD4 T cell ratio

**Fig. 1:**
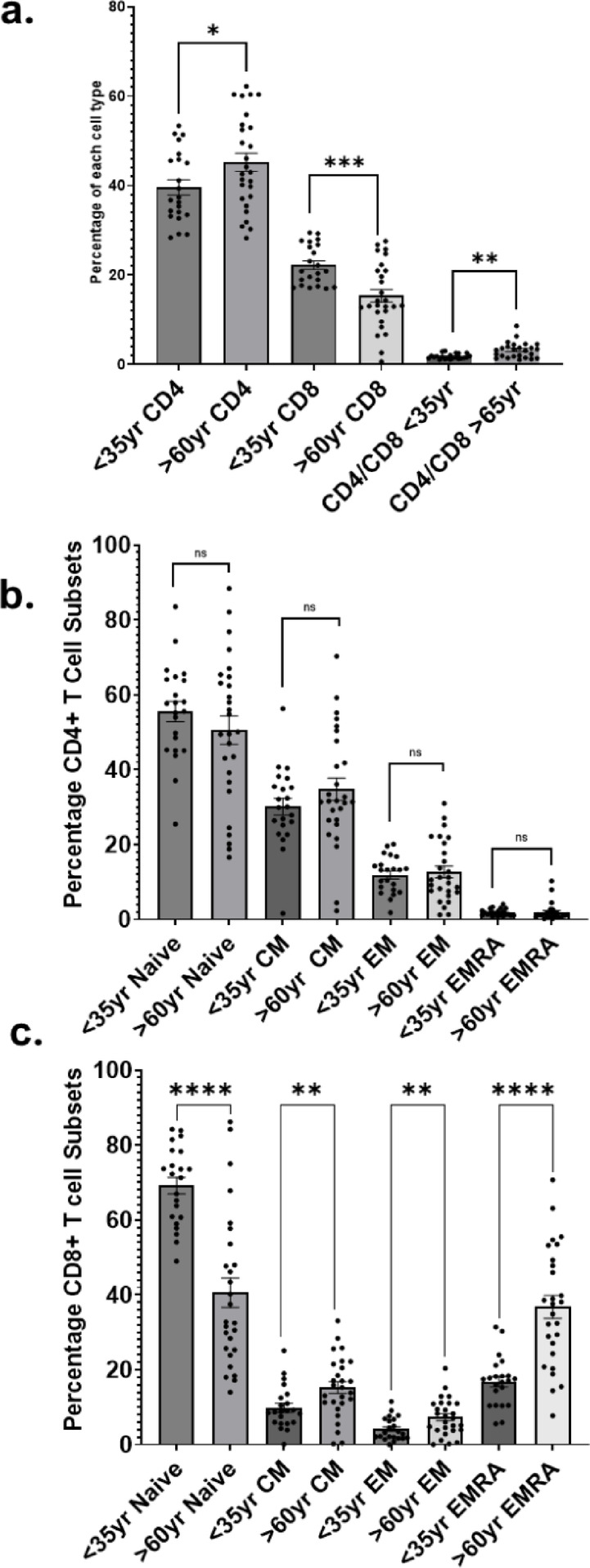
The relationship between age and T cell subtypes. (**A**) Mean CD4 + and CD8 + T cell frequencies compared between young and older participants. (**B**) CD4 + T cell subset comparisons between young and older study participants. (**C**) CD8 + T cell subset comparisons between young and older study participants. EM = effector memory. CM = central memory. EMRA = terminally differentiated effector memory cells re-expressing CD45RA. * = *p* < 0.05, ** = *p* < 0.01, *** = *p* < 0.001, ****= *p* < 0.0001.

Samples from individuals aged 60 years or older retain 31% reduction in the frequency of CD8^+^ T cells relative to samples from those aged < 35 years (*p* = 0.001). Specifically, naïve CD8 + T cells were present at only 40.6% of the T cell population in individuals aged > 60 years compared with 69.1% in those aged under 35 years (95% CI 32.51–48.66 and 64.56 to 73.78 respectively; p = < 0.0001). Effector memory CD8 + T cells were present at 7.3% of the T cell population (95% CI 5.4 to 9.3) compared with 4.1% in younger samples (95% CI 2.8 to 12.4; *p* = 0.006). Central memory CD8 + cells were present at 15.3% of the T cell population in older people (95% CI 12.1 to 18.5) compared with 9.9% (95% CI 7.4 and 12.4) in younger individuals (*p* = 0.006). Similarly, CD8 + EMRA cells were present at 36.8% (95% CI 30.5–43.1) in older people compared with 16.8% (95% CI 13.9 to 19.7) in younger individuals (p = < 0.0001). Within the CD4^+^ T cell compartment, there was no statistically significant difference between any subcategory between samples from people aged > 60 years compared with those aged < 35 years. (Supplementary Fig. 1 shows gating, Fig. 1; Table 2).

**Table 2:** Classification and quantification of T cell subsets in older (> 60 years) and younger (< 35 years) individuals. The distribution of T cell subtypes is provided below, together with estimates of variation (confidence intervals; CI) and statistical significance assessed using T tests within each T cell subset, with a statistical significance cut-off of 0.05.

T Cell Subset	Young PBMCs Mean %	Young 95% CI % Range	Old PBMCs Mean %	Old 95% CI % Range	Mean difference Young vs. Old %	*P*-value
Naïve CD4^+^	55.51	49.87–61.16	50.58	42.82–58.34	4.932	0.16
Effector Memory CD4^+^	11.92	9.771–14.07	12.69	9.466–15.91	−0.7669	0.99
Central Memory CD4^+^	30.14	25.49–34.78	34.74	28.59–40.89	−4.601	0.12
EMRA CD4^+^	1.859	1.455–2.262	1.979	1.078–2.879	−0.1199	> 0.99
Naïve CD8^+^	69.17	64.56–73.78	40.59	32.51–48.66	28.58	**< 0.0001**
Effector Memory CD8^+^	4.148	2.790–5.507	7.325	5.378–9.271	−3.176	**0.006**
Central Memory CD8^+^	9.867	7.350–12.38	15.28	12.05–18.51	−5.412	**0.006**
EMRA CD8^+^	16.81	13.88–19.74	36.80	30.55–43.06	−19.99	**< 0.0001**

#### Samples from individuals aged > 60 years demonstrate an elevated immune age compared with younger individuals

**Fig. 2:**
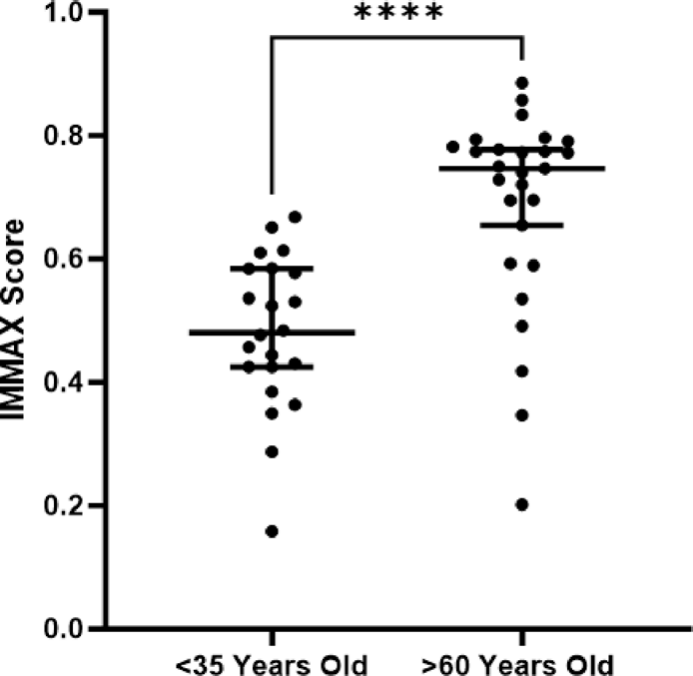
People aged > 65 years (*n* = 27) demonstrate increased IMMAX age. People aged < 35 years (*n* = 22) and > 65 years (*n* = 27) demonstrate increased IMMAX age. IMMAX age is given on the Y axis and chronological age on the X axis. Statistical significance was determined using a T test (*p* < 0.0001). The horizontal lines represent the median of the measurement. Error bars represent 95% CI (confidence interval). * = *p* < 0.05, ** = *p* < 0.01, *** = *p* < 0.001, ****= *p* < 0.0001.

The senescence status of donor T cells was assessed using an established panel of markers by flow cytometry^25^. The patterns of expression of individual senescence markers in specific T cell subpopulations is given in supplementary Fig. 2 and was used to calculate the IMMAX score. Samples from individuals aged > 60 years demonstrated a higher immune ‘age’ as calculated by the IMMAX score of 0.75 compared with 0.48 for samples from individuals aged < 35 years (p = < 0.0001; Fig. 2). This constellation of changes in older adult T cell subsets, along with differences in CD4:CD8 ratios and more defined subsets such as increased EMRA presence, indicate an aged and senescent T cell compartment amongst older adults in our study population. Individual data point dot plot visualisation for these flow markers are available in supplementary Fig. 3.

#### Samples from individuals aged > 60 years demonstrate an attenuated antibody response to recombinant SARS-CoV-2 spike protein

**Fig. 3:**
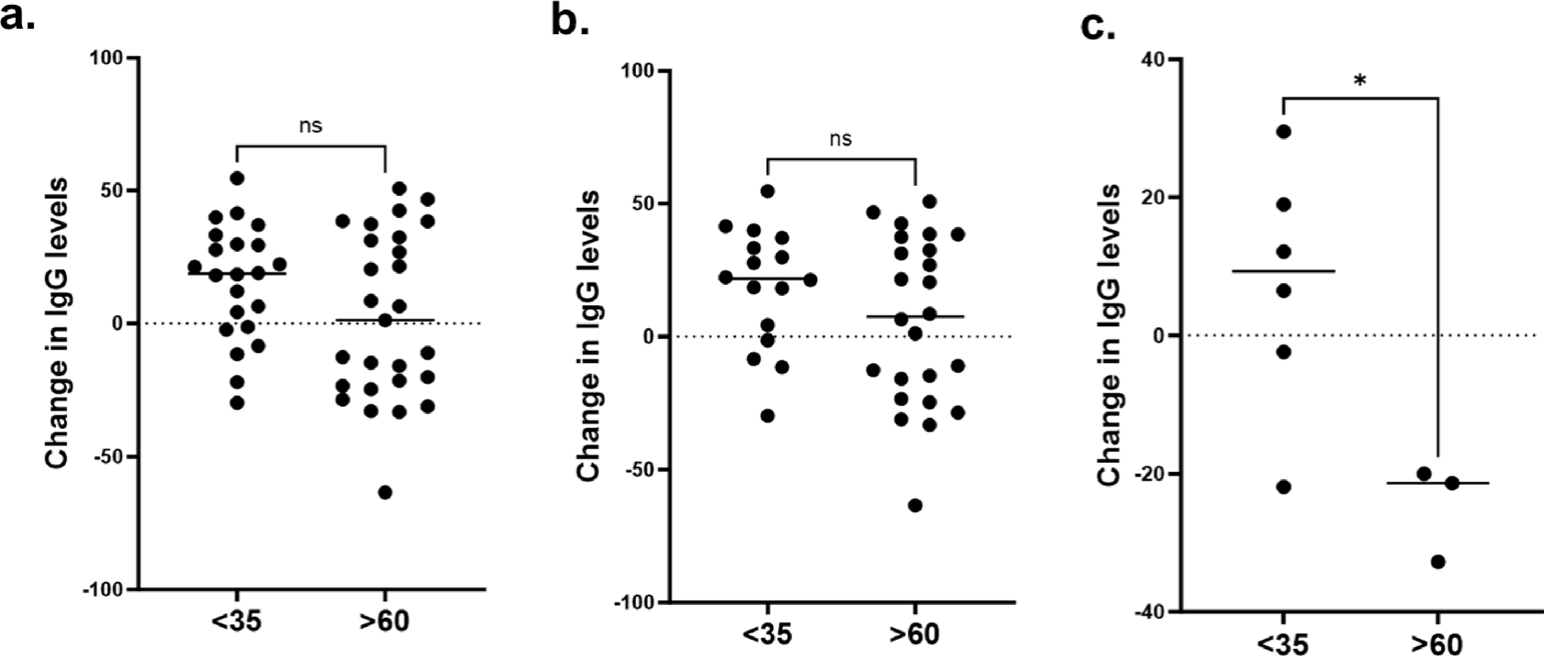
IgG production response to Spike protein in peripheral blood mononuclear cells from older and younger individuals. **a**) Change in IgG secretion in the entire study population, split by donor age. **b**) Change in IgG secretion in participants with no known diagnosis of SARS-CoV-2 or vaccination within 6 months of blood sampling **c**) Change in IgG secretion in participants with known SARS-CoV-2 infection or vaccination within 6 months of blood sampling. Change in IgG levels in response to provocation with SARS-CoV-2 spike protein are given in ng/mL of IgG for 3 technical replicates. Statistical Significance was assessed by repeated measures ANOVA. * = *p* < 0.05, ** = *p* < 0.01, *** = *p* < 0.001, ****= *p* < 0.0001.

The secretion of IgG was measured in PBMC samples from older (> 60 years) and younger (< 35 years) following challenge with recombinant Omicron B.1.1.529 SARS-CoV-2 spike protein IgG antibody secretion was used as a proxy for SARS-CoV-2 antibodies following challenge with recombinant SARS-2-CoV spike protein in vitro. In the dataset as a whole and in those with no known exposure to SARS-CoV-2 within the previous 6 months, we noted no significant change in IgG levels between older and younger participants (Fig. 3a and b). However, when we assessed individuals with known exposure to SARs-CoV-2 within the past 6 months, we identified a statistically significant increase in IgG production in the younger age group (7.15 ng/mL [SEM 7.30]) but a 24.7ng/mL (SEM 4.05) *decrease* in IgG secretion from donors aged over 60 years (*p* = 0.02; Fig. 3c). Demographic data for this stratified cohort is available within supplementary Table 3.

#### Individuals with higher immune age that have recent exposure to SARS-CoV-2 spike protein produce an attenuated antibody response

**Fig. 4:**
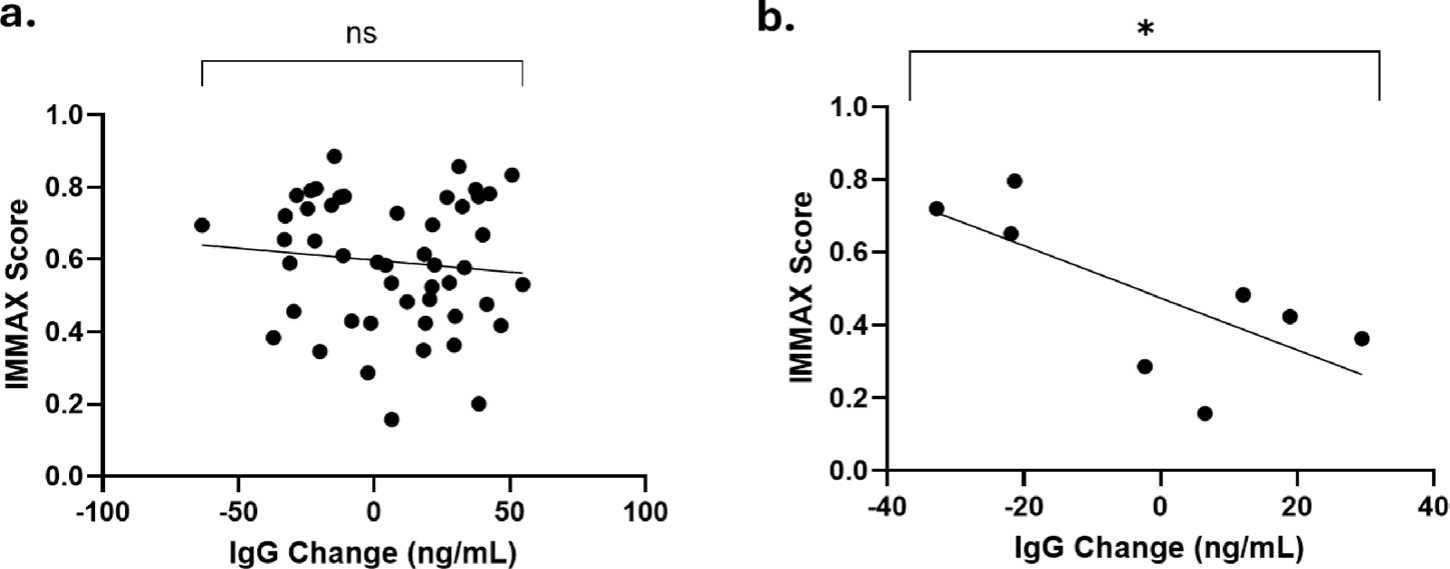
Linear regressions between IMMAX scoring and IgG production in response to spike protein. Change in IgG levels in response to provocation with SARS-CoV-2 spike protein are given in ng/mL of IgG for 3 technical replicates. Analysis was linear regressions performed between paired IMMAX scoring (y axis) and IgG production (x-axis). Circles indicate individual participant. (**A**) All Study population PBMC samples (*n* = 49). (**B**) A subset of samples from the total sample population who self-reported as diagnosed with SARS-CoV-2 infections within the 6 months leading up to the date of their blood sampling and did not have any self-reported health conditions (*n* = 8). * = *p* < 0.05, ** = *p* < 0.01, *** = *p* < 0.001, ****= *p* < 0.0001.

Biological and chronological age are not always equivalent. To determine whether cells with a higher immune age have functional deficits in terms of antibody production, we related the IMMAX age of each sample to the change in IgG production following challenge with SARS-CoV-2 by linear regression. We identified that in the full unstratified dataset, we did not see a relationship between the biological age of the immune system as measured by IMMAX and antibody production using IgG as a proxy (Fig. 4a). However, this response was attenuated by recent infection. In samples from individuals who had been exposed to SARS-CoV-2 spike protein within the last 6 months, immune age negatively correlated with IgG responsivity (b = −0.01; r^2^ = 0.51; *p* = 0.05; Fig. 4b).

#### Samples from older individuals display reduced levels of T cell receptor delta gene rearrangement

**Fig. 5:**
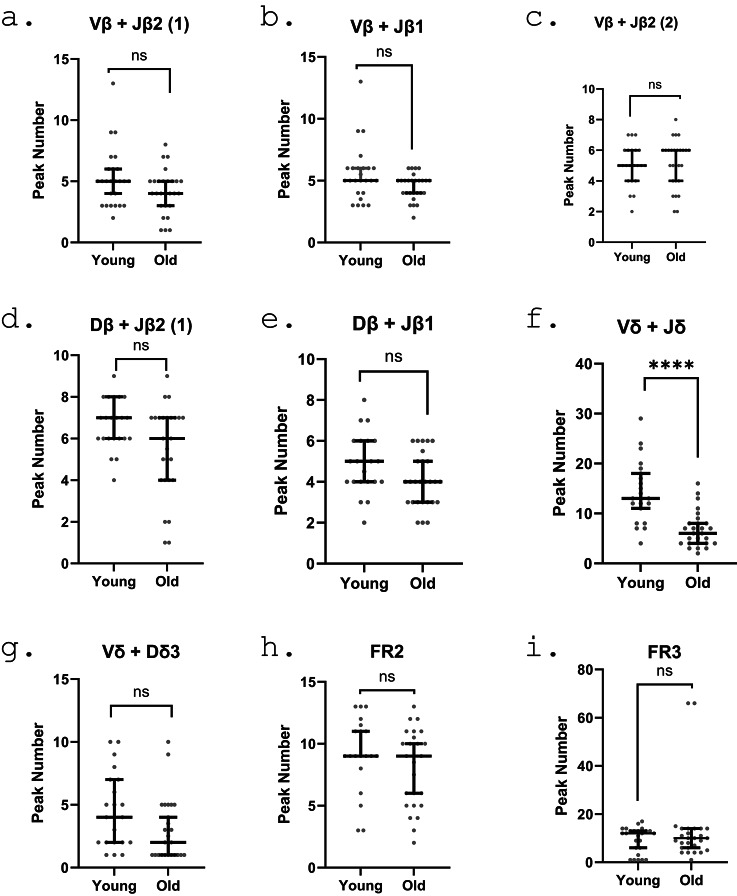
BCR and TCR gene rearrangement clonality in younger/older PBMCs. A single peak refers to a single clone within the assay, with median and 95% confidence intervals plotted. Black data points indicate individual participant median peak number for each assay. Each graph represents a different region for either BCR or TCR genes. BCR genes indicate heavy chain FR regions. TCR genes cover VDJ recombination assays. **A**) TCRβ Vβ + Jβ2 (1) assay. **B**) TCRβ Vβ + Jβ1 assay. **C**) TCRβ Vβ + Jβ2 (2) assay. **D**) TCRβ Dβ + Jβ2 (1) assay. **E**) TCRβ Dβ + Jβ1 assay. **F**) TCRδ Vδ + Jδ assay. **G**) TCRδ Vδ + Dδ3 assay. **H**) BCR FR2 assay. **I**) BCR FR3 assay. Statistical analysis performed were T test between groups. * = *p* < 0.05, ** = *p* < 0.01, *** = *p* < 0.001, ****= *p* < 0.0001.

Immunoglobulin gene and TCR gene rearrangement is an important mediator of B cell and T cell diversity. We assessed the number of the IgH immunoglobulin framework region (FR2 and FR3) gene and T cell receptor beta and delta gene rearrangements by clonality assays. We identified that whilst the majority of T cell rearrangements did not display statistically significance differences in the number of peaks identified, we did observe a reduction in the number of Vd + Jd rearrangements, with a median peak number of 13 (95% CI 11–18) in cells from younger participants compared with 6 peaks (95% CI 4–8) in untreated cells from older participants (p = < 0.0001). Similar effects were noted in samples following challenge with SARS-CoV-2 spike protein; the median peak number in cells from younger donors (< 35 years) was 15 (95% CI 9–19) compared with 6 peaks (5% CI 4–10) in cells from older participants (p = < 0.0001; Fig. 5). Other gene rearrangements tested here were not found to be significant. We identified no clear change in immunoglobulin FR2 or FR3 heavy chain rearrangement rates in cells between older and younger participants (Fig. 5). The addition of spike protein did not cause alteration of patterns of T or B cell clonality (data not shown).

## Immune age is correlated with reduced frequency of TCR delta Vd + Jd gene rearrangements

**Fig. 6:**
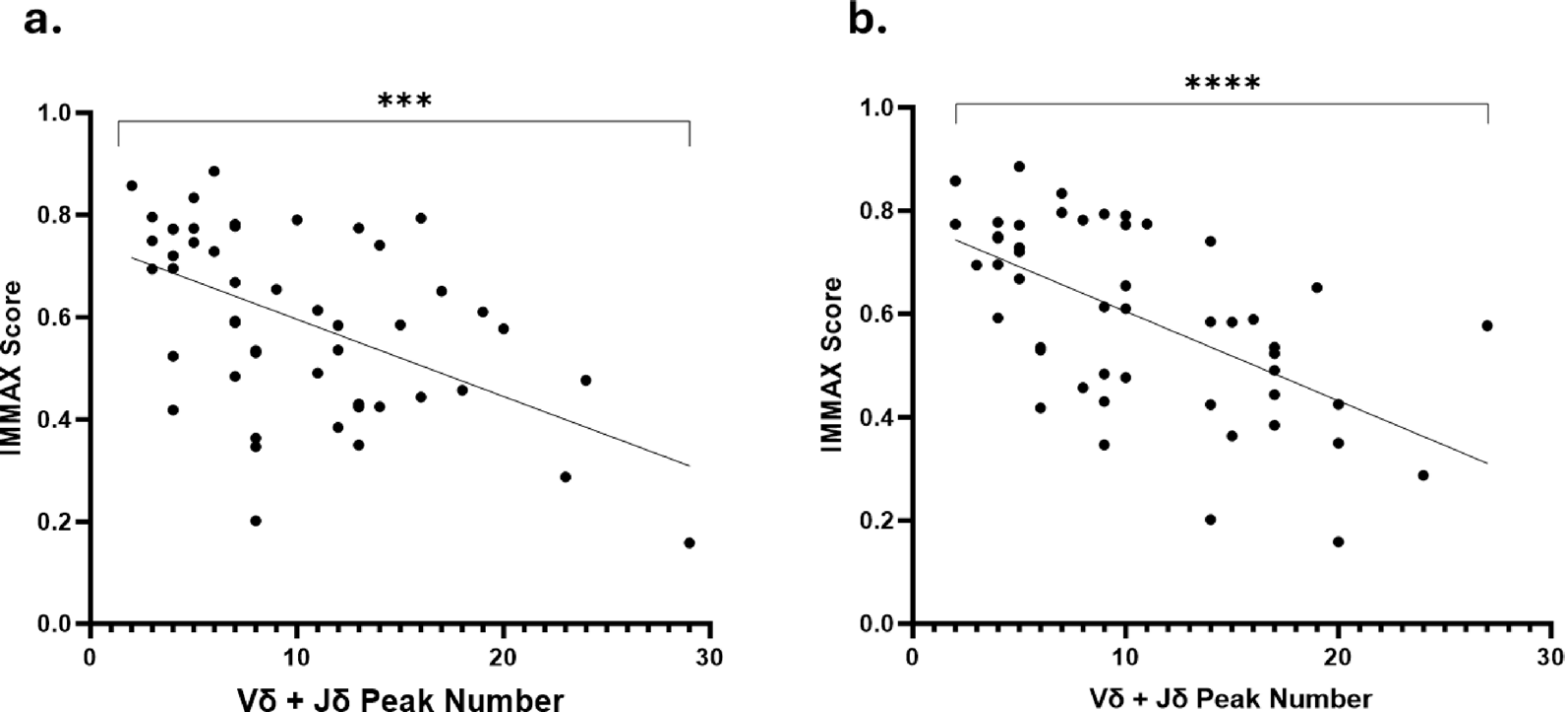
Linear regressions between IMMAX scoring and TCR delta gene clonality peak numbers. Data points indicate individual participant median peak number calculated from technical triplicates for the TCRδ Vδ + Jδ assay. Peak number for the assay is given on the x-axis and IMMAX scores given on the y-axis. Linear regressions were run for (**A**) PBMC control samples from each study participant (*p* = 0.0001, *n* = 49) and (**B**) PBMC samples from each participant treated with spike protein (*p* < 0.0001, *n* = 49). * = *p* < 0.05, ** = *p* < 0.01, *** = *p* < 0.001, ****= *p* < 0.0001.

We related the immune age of each sample to the frequency of TCR delta Vδ + Jδ gene rearrangements with or without SARS-CoV-2 spike protein challenge by linear regression. We identified that higher IMMAX scores were negatively correlated with the number of TCR delta Vδ + Jδ recombination peaks regardless of exposure to SARS-CoV-2; b = −0.02; r^2^ = 0.23; p = < 0.0001 for unexposed individuals and b = −0.02; r^2^ = 0.0.35; p = < 0.0001 following SARS-CoV-2 challenge respectively (Fig. 6a and b respectively).

## Discussion

As we age, the immune system becomes less diverse and less able to mount appropriate responses to infectious agents or to vaccination. We aimed to assess the relationship between immune age and immune cell proportions in the context of response to recombinant SARS-CoV-2 (omicron variant) antigen challenge. We also assessed the relationship between immune age and IgH or T cell receptor clonal diversity. We have demonstrated that older individuals demonstrate characteristic changes in the composition of their T cell pool and altered cell surface marker expression consistent with cellular senescence. Furthermore, we present evidence that a higher immunological age in isolated peripheral blood mononuclear cells from older individuals is associated with compromised ability to secrete IgG, if participants had been exposed to SARS-CoV-2 in the past 6 months. This finding suggests that older individuals who have been recently exposed to SARS-CoV-2 antigen may demonstrate a reduced antibody response to subsequent challenge with SARS-CoV-2 within this timeframe. We also demonstrate that immune age is correlated with decreased T cell receptor gamma and delta immunoglobulin gene rearrangements. which is an important contributor to the diversity of T cell and B cell responses. TCR gd rearrangements are common in the T cells resident in the gut, which is known to become more permeable with advancing age^26^. This may have implications for the breadth of immune response that can be mounted in response to antigen challenge, which influences not just response to infectious agents, but also immunosurveillance and capacity for clearance of senescent cells.

Our data agree with previous reports of changes in immune cell subsets and increasing immunosenescence with age^27^. Increased CD8^+^ and CD57^+^ expression in naïve T cells has been previously demonstrated to characterise functional and replicative senescence prior to terminal differentiation. CD57 expressing T cells have been shown to possess shortened telomeres; although other T cell subtypes are also more likely to have shorter telomeres, indicating stress induced senescence may be more relevant to T cell dysfunction than replicative senescence^28^. Loss of CD28 and CCR7 is considered part of a senescent phenotype due to inadequate maturation and selection of naïve and memory CD8^+^ T cells^29^. The loss of NKG2D markers from naïve CD8^+^ T cells in older adults also suggests a picture of reduced responsiveness to viral infection with age. Evidence of CD8 compartment dysfunction is also suggested by the upregulation of the late immune checkpoint marker NKG2 A in older individuals which indicates repeated stimulations and replicative senescence within aged CD8^+^ T cells^20,30^.

We also demonstrate a link between immunosenescence and reduced IgG response to SARS-CoV-2 antigen challenge in samples from older participants, which is most marked in older individuals with recent prior exposure to SARS-CoV-2. Antibody titres may be attenuated in elderly individuals due to ineffective cellular communication and poor subsequent activation of adaptive immunity. Recent exposure to SARS-CoV spike protein may attenuate responses to future challenge with SARS-CoV-2. Optimum response to SARS-CoV-2 vaccination in this age group may therefore not be observed within 6 months of recent infection and better responses may be achieved after the 6-month window. Caution should be taken in interpreting these data in a real world setting however, since in vivo infection to SARS-CoV-2 involves exposure to epitopes other than spike. Our data also cannot be extrapolated to other infectious agents, since our data were drawn solely from isolated cells in culture, without the presence of other components of the immune system, following challenge with spike protein alone. Similarly, our study did not include number of medications to which our participants had been exposed to, but this has been demonstrated to have little impact on immune cell senescence^31^.

We also provide evidence to support previous observations of reduced T cell diversity with age, regardless of spike antigen exposure. We next sought to provide evidence to support previous observations of reduced T cell diversity with age. The primary category of rearrangements we observed involved T cell receptor delta. This is noteworthy, since others have reported changes in the TCR alpha-beta TCR repertoire^32^. TCR delta rearrangements are however known to occur with reduced frequency with age and are thought to transfer information between different immune compartments^33^. TCR delta excision (TREC) circle levels are also a marker for active thymopoiesis^34^. TCR delta rearrangement has also been described to precede alpha-beta rearrangement and to be an essential part in production of diverse TCR alpha repertoire in mouse^35^. It has also been demonstrated to be the default pathway in human thymocyte development^36^. Our observation of reduced TCR delta rearrangement may therefore be a reflection of the thymic changes that characterises the reduced immunological tolerance and immunity of ageing. Gamma/delta T cells play an important role in enabling B cell antibody production^37,38^. It is important to state that we did not expect to see changes in TCR diversity because of experimental intervention conducted in this study over the timescales studied, and accordingly we did not observe this.

There is evidence that the telomere shortening that occurs in replicatively-senescent cells may impede T cell clonal expansion in response to viral infection and lead to lymphopenia in aged individuals^39^. Reduced plasticity for gene recombination may be influenced by senescence of the cells in question but may also contribute to the accumulation of senescent cells by impairment of clearance^40^. Previous reports suggest that COVID-19 disease severity is linked within poorer TCR diversity^41^. It is worth noting that to experimentally validate the clonal changes across age demonstrated in this paper regarding gamma/delta T cells, full sequencing would be appropriate.

It should be noted that our results are drawn from a simplified in vitro system, that cannot capture the complexities of exposure to infectious agents or vaccination in a complex living system, with both innate and adaptive immune systems and exposure to multiple epitopes. Furthermore, we have not measured antibody secretion directly, instead relying on a proxy marker, IgG. Immunoglobulin G (IgG) represents approximately 75% of antibody content^42^, so in the absence of access to an animal-free SARS-CoV-2 specific ELISA, IgG was determined to be an appropriate proxy, given the fact that the cells were maintained in a controlled environment and were exposed only to SARS-CoV-2 antigen. Future work might benefit from using a specific neutralising antibody assay to determine immune efficacy. There are also some differences in the demographics and COVID history between our older and younger samples. This could have introduced some biases. The samples were taken from community dwelling older and younger people however and do represent real world differences in demographics in our cross-sectional study population.

Immunosenescence is thought to be a driver not just of reduced responses to infection or vaccination, but also of impaired systematic clearance of other emerging senescent cell compartments. The work described here provides evidence that increasing immune biological ‘age’ is associated with reduction in the magnitude and diversity of immune responses following challenge with recombinant SARS-CoV-2 spike protein. Although we have not assessed the transferability of our finding to other infectious agents, and additional work to assess these parameters in an in vivo setting with a more complex immune challenge, our data suggest that immune senescence may represent a promising future therapeutic target to improve response to vaccination and outcomes of infectious disease in the older population.

## Electronic supplementary material

Below is the link to the electronic supplementary material.

Supplementary Material 1

Supplementary Material 2

## Data Availability

All data generated or analysed during this study are included in this published article (and its Supplementary Information files). Additional access to raw data files is available from the corresponding author on reasonable request.
