# Supplementary material for: Immune age is correlated with decreased TCR clonal diversity and antibody response to SARS-CoV-2
Source: Sci Rep. 2025 Jun 6;15:19883. doi: 10.1038/s41598-025-04736-4 (PMC12144168; doi:10.1038/s41598-025-04736-4)
Supplement: Supplementary file 1 — Supplementary Material 1 [file 41598_2025_4736_MOESM1_ESM.docx]

**Supplementary table 1: Antibody details for characterisation of cellular immune age.** The details of the antibodies and dilution factors used for flow cytometry classification of IMMAX immune age are given below.

| **Target** | **Intra/extracellular** | **Fluorochromes** | **Company** | **Dilution** |
| --- | --- | --- | --- | --- |
| Viability | Live/dead cell stain | 405/520 | Miltenyi | 1/100 |
| CD4 | Extracellular | Brilliant Violet 650 | Biolegend | 1/50 |
| CD8 | Extracellular | APC-Vio770 | Miltenyi | 1/50 |
| CCR7 | Extracellular | PerCP-Vio700 | Miltenyi | 1/50 |
| CD27 | Extracellular | Brilliant Violet 605 | Biolegend | 1/50 |
| CD28 | Extracellular | Brilliant Violet 785 | Biolegend | 1/50 |
| CD45RA | Extracellular | VioBright R720 | Miltenyi | 1/25 |
| CD57 | Extracellular | Brilliant Violet 711 | Biolegend | 1/50 |
| KLRG1 | Extracellular | APC | Miltenyi | 1/50 |
| NKG2A | Extracellular | VioBright 515 | Miltenyi | 1/50 |
| NKG2D | Extracellular | PE-Vio615 | Miltenyi | 1/50 |
| FOXP3 | Intracellular | PE | Miltenyi | 1/50 |
| CD24 | Extracellular | PE-Vio770 | Miltenyi | 1/50 |
| CD25 | Extracellular | VioBrightV423 | Miltenyi | 1/25 |

**Supplementary table 2: FR2 and FR3 B cell clonality primer sequences.** Details of primer sequences used for B Cell clonality multiplex PCR microsatellite data generation.

| **FR2** |  |  |
| --- | --- | --- |
| VH1-FR2 (1-2) | CTGGGTGCGACAGGCCCCTGGACAA | JJM van Dongen *et al.(2003) Leukemia 17; 2257–2317* |
| VH2-FR2 (2-5) | TGGTATCCGTCAGCCCCCAGGGAAGG | JJM van Dongen *et al.(2003) Leukemia 17; 2257–2317* |
| VH3-FR2 (3-7) | GGTCCGCCAGGCTCCAGGGAA | JJM van Dongen *et al.(2003) Leukemia 17; 2257–2317* |
| VH4-FR2 (4-4) | TGGATCCGCCAGCCCCCAGGGAAGG | JJM van Dongen *et al.(2003) Leukemia 17; 2257–2317* |
| VH5-FR2 (5-51) | GGGTGCGCCAGATGCCCGGGAAAGG | JJM van Dongen *et al.(2003) Leukemia 17; 2257–2317* |
| VH6-FR2 (6-1) | TGGATCAGGCAGTCCCCATCGAGAG | JJM van Dongen *et al.(2003) Leukemia 17; 2257–2317* |
| VH7-FR2 (7) | TTGGGTGCGACAGGCCCCTGGACAA | JJM van Dongen *et al.(2003) Leukemia 17; 2257–2317* |
| **FR3** |  | JJM van Dongen *et al.(2003) Leukemia 17; 2257–2317* |
| VH1-FR3 (1-2) | TGGAGCTGAGCAGCCTGAGATCTGA | JJM van Dongen *et al.(2003) Leukemia 17; 2257–2317* |
| VH2-FR3 (2-5) | CAATGACCAACATGGACCCTGTGGA | JJM van Dongen *et al.(2003) Leukemia 17; 2257–2317* |
| VH3-FR3 (3-7) | TCTGCAAATGAACAGCCTGAGAGCC | JJM van Dongen *et al.(2003) Leukemia 17; 2257–2317* |
| VH4-FR3 (4-4) | GAGCTCTGTGACCGCCGCGGACACG | JJM van Dongen *et al.(2003) Leukemia 17; 2257–2317* |
| VH5-FR3 (5-51) | CAGCACCGCCTACCTGCAGTGGAGC | JJM van Dongen *et al.(2003) Leukemia 17; 2257–2317* |
| VH6-FR3 (6-1) | GTTCTCCCTGCAGCTGAACTCTGTTG | JJM van Dongen *et al.(2003) Leukemia 17; 2257–2317* |
| VH7-FR3 (7) | CAGCACGGCATATCTGCAGATCAG | JJM van Dongen *et al.(2003) Leukemia 17; 2257–2317* |

**Supplementary table 3: Stratified study cohort who experienced positive COVID-19 tests within 6 months prior to PBMC sampling.** Demographic details of the individuals within the cohort who reported a positive test date within 6 months prior to PBMC sampling. All community living adults also reported no current health conditions, long term chronic conditions or immunological conditions of any severity.

| Patient characteristics (N=9) | |
| --- | --- |
| Number of adults aged 35 or below | 6 |
| Number of adults aged 65 or above | 3 |
| % Male (35&U) | 50% |
| % Male (60+) | 0% |
| Mean Age (35&U) | 28 |
| Mean Age (60+) | 74 |
| % Vaccinated against SARS-CoV-2 (35&U) | 100% |
| % Vaccinated against SARS-CoV-2 (60+) | 100% |
| Incidents of severe COVID-19 disease (35&U) | 0 |
| Incidents of severe COVID-19 disease (60+) | 0 |
