# Supplementary material for: Immune age is correlated with decreased TCR clonal diversity and antibody response to SARS-CoV-2
Source: Sci Rep. 2025 Jun 6;15:19883. doi: 10.1038/s41598-025-04736-4 (PMC12144168; doi:10.1038/s41598-025-04736-4)
Supplement: Supplementary file 2 — Supplementary Material 2 [file 41598_2025_4736_MOESM2_ESM.docx]

**A**

CD4

CD8

**B**


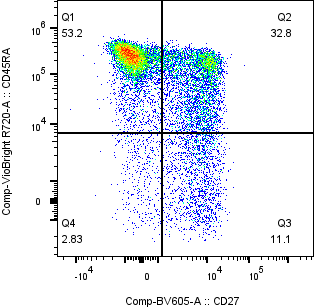

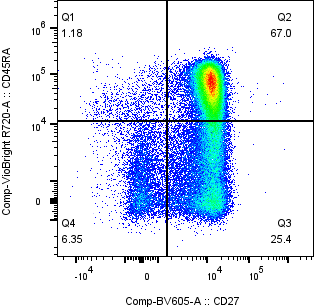

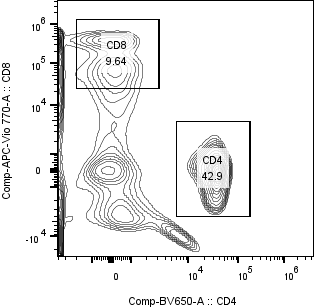

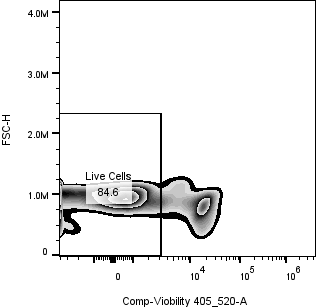

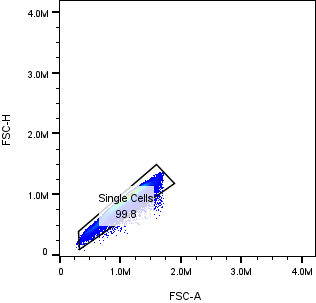

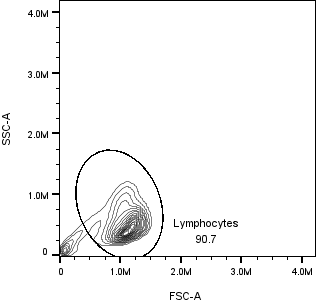


CD4

CD8

Supplementary figure 1: Gating strategies and T cell subset comparisons between young and older adults. A) Demonstrates a step-by-step approach to gating for <35 year old samples. B) Demonstrates a step-by-step approach to gating for >60 year old samples. Gating strategies for the 4 major subgroups of CD4+ and CD8+ T cells are presented, which were applied to all 49 study participant samples (Q1: EMRA T cells, Q2: Naïve, Q3: Central Memory, Q4: Effector Memory). EM = effector memory. CM = central memory. EMRA= terminally differentiated effector memory cells re-expressing CD45RA.

Supplementary figure 2: Changes in cell surface marker expression across T cell sub compartments. Heatmap shows the mean changes for aged adults (60+ vs <35 years old) in the frequencies of specific T cell markers which indicate senescence for CD4 and CD8 T cells. Red indicates increasing frequencies and green indicates decreases. Statistically significant changes are highlighted with a white box. Each markers analysis was carried out separately using unpaired T tests.

**A**


Supplementary figure 3: Changes in cell surface marker expression across T cell sub compartments A) CD4 & B) CD8. Dot plots show the mean and granular changes for aged adults (60+ vs <35 years old) in the frequencies of specific T cell markers which indicate senescence for CD4 and CD8 T cells. A) markers in sub-compartment CD4 and B) markers in sub-compartment CD8. Statistically significant changes are signified with stars between younger and older cohorts. Each markers analysis was carried out separately using unpaired T tests.

**B**
